# Sleep onset (mis)perception in relation to sleep fragmentation, time estimation and pre-sleep arousal

**DOI:** 10.1016/j.sleepx.2020.100014

**Published:** 2020-03-28

**Authors:** Lieke W.A. Hermans, Marina M. Nano, Tim R. Leufkens, Merel M. van Gilst, Sebastiaan Overeem

**Affiliations:** a Department of Electrical Engineering, Eindhoven University of Technology, De Zaale, Eindhoven, the Netherlands; b Philips Research, High Tech Campus 34, Eindhoven, the Netherlands; c Center for Sleep Medicine Kempenhaeghe, Sterkselseweg 65, Heeze, the Netherlands

**Keywords:** Insomnia, Sleep state misperception, Sleep onset latency, Sleep fragmentation, Time estimation, Pre sleep arousal

## Abstract

**Study objective:**

To elucidate the contribution of time estimation and pre sleep arousal to the component of sleep onset misperception not explained by sleep fragmentation.

**Methods:**

At-home ambulatory polysomnograms (PSGs) of 31 people with insomnia were recorded. Participants performed a time estimation task and completed the Pre Sleep Arousal Scale (PSAS). Based on previous modelling of the relationship between objectively measured sleep fragmentation and sleep onset misperception, the subjective sleep onset was estimated for each participant as the start of the first uninterrupted sleep bout longer than 30 min. Subsequently, the component of misperception not explained by sleep fragmentation was calculated as the residual error between estimated sleep onset and perceived sleep onset. This residual error was correlated with individual time estimation task results and PSAS scores.

**Results:**

A negative correlation between time estimation task results and the residual error of the sleep onset model was found, indicating that participants who overestimated a time interval during the day also overestimated their sleep onset latency (SOL). No correlation was found between PSAS scores and residual error.

**Conclusions:**

Interindividual variations of sleep architecture possibly obscure the correlation of sleep onset misperception with time estimation and pre sleep arousal, especially in small groups. Therefore, we used a previously proposed model to account for the influence of sleep fragmentation. Results indicate that time estimation is associated with sleep onset misperception. Since sleep onset misperception appears to be a general characteristic of insomnia, understanding the underlying mechanisms is probably important for understanding and treating insomnia.

## Highlights

• Sleep onset misperception is probably a multifactorial process.
• We identify the part of misperception not explained by fragmentation.
• This metric was correlated with the time estimation and pre sleep arousal.
• Results indicate that time estimation is involved in sleep onset misperception.

## 1 Introduction

Many people with insomnia overestimate their sleep onset latency (SOL) and underestimate their total sleep time (TST) compared to objective sleep recordings [1,2]. This is referred to as sleep state misperception. The underlying mechanisms of sleep state misperception remain to be elucidated [3]. For sleep onset misperception, multiple factors have been proposed to play a role, including sleep fragmentation, an altered time estimation ability and pre sleep arousal [3].

Time estimation has been hypothesized to be associated with sleep onset misperception. The underlying idea is that people who overestimate time intervals during the day are thought to also overestimate their time awake in bed [[3], [4], [5]]. Time estimation in insomnia was tested in three studies, comparing patients with insomnia to healthy controls, using various time estimation paradigms [[4], [5], [6]]. However, none of these studies found a significant difference between the time estimation ability of insomnia patients and healthy controls [[4], [5], [6]]. In addition, Rioux et al., reported that they did not find a correlation between time estimation and the severity of insomnia [5]. These findings led Harvey and Tang to conclude in their review that the hypothesis of a time estimation deficit in insomnia has negative evidence of moderate quality [3]. However, not all patients with insomnia misperceive their sleep, and severe complaints of insomnia do not necessarily co-occur with severe sleep state misperception. Therefore, it could be argued that a better approach would be to take into account the actual discrepancy between objective and subjective sleep when assessing the influence of time estimation [5]. In other words, if time estimation is an underlying mechanism of sleep onset misperception, it is plausible that the ability of an individual to estimate time is correlated with that individual's amount of sleep misperception, rather than the seriousness of the insomnia complaints. Thus, in general, not taking the amount of sleep misperception into account as an outcome variable could result in overlooking relevant contributing factors. This probably also applies for factors other than time estimation.

Increased pre sleep arousal is found in approximately 40% of insomnia patients [7]. It has been hypothesized that worrying before falling asleep can lead to an overestimation of the SOL [3,8]. This hypothesis was based on the fact that psychological distress can cause a magnification of complaints in somatic and psychiatric disorders [9]. Additionally, results of early research show that people estimate elapsed time as longer when they have to process more information [10]. Indeed, a positive correlation between subjective SOL and pre sleep arousal assessed with an interview was found in 34 subjects, of which 13 had complaints of insomnia [11]. In the same study, no correlation between pre sleep arousal and objective SOL was found [11]. Since an increased pre sleep arousal influenced the perception of the sleep onset without altering objective SOL [11], it seems likely that pre sleep arousal influences the amount of sleep onset misperception. However, this hypothesis has not been confirmed.

Correlating time estimation and pre sleep arousal with the amount of sleep state misperception is challenging, because sleep state misperception is most probably a multifactorial process, which is also influenced by objective characteristics of sleep [3,12,13]. Recently, we quantitatively modelled the relationship between sleep fragmentation and sleep onset misperception [14]. We identified sleep fragmentation as the most important objectively measurable characteristic influencing sleep onset misperception [14]. This conclusion fits with previous research, where the sense of being asleep prior to awakening from NREM sleep was shown to depend on the length of the preceding uninterrupted sleep fragment [[15], [16], [17]]. Thus, a certain minimum amount of continuous sleep seems to be required for people to recall falling asleep. It is possible that large interindividual variations of sleep architecture obscure the correlation of the amount of sleep onset misperception with time estimation and pre sleep arousal. This could especially be important in small groups of participants.

In previous work, we modelled perceived sleep onset as a function of the minimum length that an uninterrupted sleep fragment requires to be perceived as sleep [14]. In the model, it was assumed that sleep fragments at sleep onset are not perceived as sleep if they are interrupted too soon [14]. In a follow up study, we applied this so-called sleep length model in a larger group of 139 people with insomnia with various degrees of sleep state misperception, and 93 healthy controls [18]. We also calculated optimum model parameters for individual participants. For these individual optimal parameter we proposed the name Sleep Fragment Perception Index (SFPI) [18]. Comparing SFPIs on the group level showed significant differences between participants with and without sleep onset misperception [18]. Furthermore, the model did not fully predict the amount of sleep onset misperception based on sleep fragmentation only. This supports the notion that sleep misperception is multifactorial.

We hypothesize that the predictive ability of the sleep length model in individual study participants could be applied to identify other contributing factors. For example, if a participant has more sleep onset misperception than could be expected from sleep architecture alone, it is likely that other factors play a role. Thus, a correlation with the prediction error of the model could indicate such factors, including an influence of pre sleep arousal and time estimation. In this study, we aim to elucidate the contribution of time estimation and pre sleep arousal to sleep onset misperception, by specifically assessing the correlation with the component of sleep onset misperception not explained by sleep fragmentation. This approach enables us to take into account interindividual variations of sleep architecture.

## 2 Methods

### 2.1 Participants

Data for this study were collected as part of a prospective study of sleep architecture in people with insomnia. We analyzed ambulatory PSG recordings of 31 participants with insomnia, who were on the waiting list of the Kempenhaeghe Center for Sleep Medicine to receive cognitive behavioral therapy for insomnia (CBT-I). Subjects were included if a complete sleep diary was available. To make sure that the objective sleep onset was recorded in all participants, PSG recordings were excluded when starting later then the lights off time reported by the participant or when recording started with an epoch scored as sleep. In order to be eligible to participate, subjects had to meet the following criteria: age older than 18, a diagnosis of insomnia according to DSM-IV criteria and sleep medication use less than 3 times per week. Exclusion criteria were pregnancy, conditions preventing taking part in neuropsychological tests, patients who lack the functional capacity to provide informed consent and patients who are not able to adhere to the study protocol.

The study was conducted in accordance with the code of ethics on human experimentation established by the World Medical Association's Declaration of Helsinki (1964) and amended in Edinburgh (2000). The study protocol (W17.043) was approved by the medical ethics committee of Maxima Medical Center, Veldhoven, the Netherlands. All subjects provided written informed consent.

### 2.2 Study design

The measurements consisted of one night of ambulatory PSG at home. Electrodes were attached between 19:30 and 21:30 in the evening of the PSG night and participants were free to choose their own bedtimes. Participants were asked to not take occasionally-used psychoactive drugs whose primary function is to induce sleep, including over-the-counter-available melatonin, from one week preceding the sleep measurement night until the night of the measurements. Coffee and alcohol were prohibited on the day preceding the PSG recording. One week before the night of the sleep recording, an additional appointment was scheduled to perform a time estimation task and complete several questionnaires.

### 2.3 Measurements

PSG - A six-channel electroencephalogram (C3, C4, F3, F4, O1, O2), electrooculogram (ECG) and electromyogram were performed, using a Natus Embletta MPR recorder, interfaced with a ST + Proxy. Additionally, ECG, abdominal and thoracic respiration effort, SpO_2_ from finger pulse-oximetry and body position and activity were recorded. Visual sleep staging for all recordings was performed according to American Academy of Sleep Medicine (AASM) criteria by an experienced somnotechnologist.

Electronic sleep diary – At the morning after the sleep recording, participants completed an electronic version of the consensus sleep diary [19].

Time estimation task - During the time estimation task, subjects were asked to indicate the end of a 10-min waiting period by pressing a key on a laptop. We chose to ask the participants to press a key, which is similar to the study design of Harrow et al. [4], rather than asking participants to estimate the length of a fixed time interval. This approach has the advantage of preventing people's tendency to call rounded numbers [4]. We chose a time interval of 10 min, because this time interval is probably long enough to resemble the actual situation when lying in bed. Before the start of the test, patients were asked to cover all clocks in the room and to sit back and relax. The time estimation tasks were performed at the participant's home, under supervision of a researcher. Time estimation tasks were not performed on a fixed time during the day. All time estimation tasks were performed between 10:00 in the morning and 19:30 in the evening. The output of the time estimation task was the amount of seconds elapsed before pressing the key. Thus, a number of less than 600 s could be interpreted as an overestimation of elapsed time (eg, the participant experiences an elapsed time of 10 min) while in reality the elapsed time was shorter.

PSAS – Participants completed the PSAS [20] to indicate arousal prior to falling asleep. A higher score on the PSAS indicates more pre sleep arousal. Scores for the somatic and cognitive subscales were combined into one total score. This total score was used for further analysis.

ISI – Participants completed the Insomnia Severity Index (ISI) [21] to indicate the severity of their insomnia complaints. The results of the ISI questionnaire were used to verify that all participants had at least subthreshold insomnia, indicated by an ISI score of at least eight.

### 2.4 Data analysis - sleep length model

**Fig. 1:**
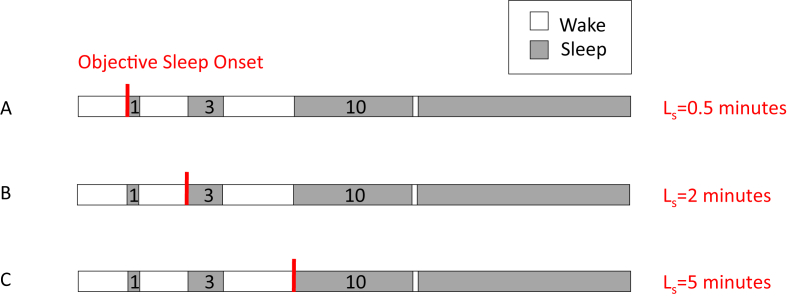
Sleep Length Model. The estimated sleep onset changes depending on the independent parameter of the model, which was defined as the start of the first continuous sleep fragment longer than L minutes, with L varying from 0.5 to 40. In (A) we assume that sleep fragments with a length below 30 s are not perceived as sleep. In this case, the sleep onset from the model is the same as the objective sleep onset according to the AASM definition. (B) If we assume that sleep fragments with a length below 2 min are not perceived as sleep, the estimated sleep onset shifts to the second sleep bout. (C) If we assume that sleep fragments with a length below 5 min are not perceived as sleep, the sleep onset shifts past the two shorter sleep bouts. Reprinted with permission from: Hermans LWA, Leufkens TR, van Gilst MM, et al. Sleep EEG characteristics associated with sleep onset misperception. Sleep Med. 2019; 57. https://doi.org/10.1016/j.sleep.2019.01.031.

**Fig. 2:**
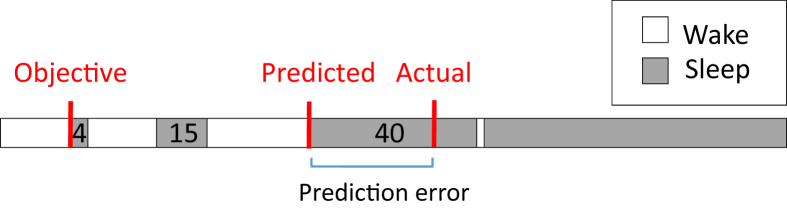
Hypothetical example of sleep onset misperception in an individual. We assigned a Sleep Fragment Perception Index (SFPI) of 30 min to each individual. From this assumption, the predicted sleep onset is the start of the first uninterrupted sleep fragment longer than 30 min. The actual perceived sleep onset is the subjective sleep onset of the participant, as obtained from the consensus sleep diary. The residual error, which can be viewed as the part of sleep onset misperception not explained by sleep fragmentation, is the difference between predicted sleep onset and actual sleep onset, indicated by a blue line. In this individual, after taking into account the presumed influence of sleep fragmentation, the Sleep Onset Latency (SOL) is still overestimated. Therefore, one could hypothesize that either poor time estimation or high pre sleep arousal plays a role in this situation.

In the sleep length model, it was assumed that sleep bouts with insufficient length at sleep onset are perceived as wake [14]. Thus, it was assumed that sleep onset was perceived as the start of the first sleep fragment longer than L minutes. Sleep length parameter L was the independent parameter of the model (ie, the length a continuous sleep fragment should have in order to be perceived as sleep). Any wake fragment of at least one 30s epoch was considered as an interruption of sleep. This procedure is illustrated in Fig. 1. In a previous study, we varied parameter L from 0.5 to 60 min in a cohort of 139 people with insomnia and 92 healthy controls to test different model assumptions [18]. We found a median optimal parameter L of approximately 30–35 min for participants with insomnia, with small differences depending on the exact criteria of the subgroup that was selected [18]. The optimal parameter L was referred to as SFPI. In the current study, we assigned the same reference SFPI of 30 min to all participants and used the model to estimate perceived sleep onset for each participant as the start of the first uninterrupted sleep fragment longer than 30 min. This is illustrated in Fig. 2. Subsequently, the residual error between estimated sleep onset and actual perceived sleep onset was calculated. This residual error was referred to as ‘sleep onset misperception not explained by sleep fragmentation’.

### 2.5 Statistical analysis

All outcomes were reported as mean ± standard deviation (sd) unless stated otherwise. The residual error of the sleep length model was correlated with the results of the time estimation task and the PSAS. Participants tended to round off their subjective SOL to 10 or 15 min.

In total, 14 participants reported their subjective SOL as a multiplicity of 15 min, and all but one participant reported SOL as a multiplicity of five min. Therefore, the SOLs from the consensus sleep diary were considered categorical variables. Because these subjective SOLs were used for the calculation of the residual error of the model, all correlation with this variable were assessed using Spearman's correlation test. Spearman's correlation test was also used in case of non-linearity of the other variables.

## 3 Results

### 3.1 Demographics and sleep characteristics

Sleep was recorded in 31 participants (14M, 17F, age 50.8 ± 15.1 [range 18–71]). Participants had an ISI score of 17.9 ± 3.5 [range 9 21]. Time estimation task results were available in 29 participants and PSAS scores were available in 27 participants. The objective SOL was 18.1 ± 25.3 [0–112] minutes and the subjective SOL from the consensus sleep diary was 31.4 ± 35.8 [5–165] minutes. The amount of SOL misperception was 13.4 ± 31.4 [-53 - 145]. Five participants underestimated their SOL. The amount of SOL misperception was not correlated with the ISI scores (Pearson r = −0.01, p = 0.97).

Two participants had a very large subjective SOLs compared to the rest of the group (subjective SOL of 165 and 150 min; in both cases > mean + 3 sd) and therefore were considered outliers. These participants were males of 50 and 55 years old, who did not have any relevant comorbidities listed. We did not a priori exclude these participants from analysis.

### 3.2 Time estimation task

**Fig. 3:**
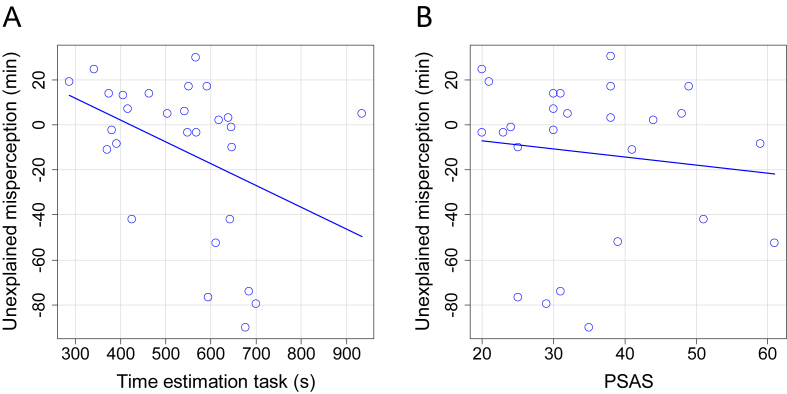
Correlations of the residual error from the sleep length model. (A) Correlation of residual error with time estimation task results (n = 29; Spearman rho = −0.50, p = 0.007). (B) Correlation of residual error with Pre Sleep Arousal Scale scores (n = 27; Spearman rho = −0.13, p = 0.53).

The average score on the time estimation task was 548 ± 139 [371–935] seconds. A negative correlation was found between time estimation task results and the residual error of the sleep length model (Spearman rho = −0.50, p = 0.007; Fig. 3a). It should be noted that a time estimation task score lower than 600 s indicates an overestimation of elapsed time. For example, if a participant indicated that the 10 min interval had passed after nine min, this indicates an overestimation of the nine-min time interval with one min. When assessing the same correlation again but without the two outliers with a very large subjective SOL, still a significant negative correlation was found (Spearman rho = −0.45, p = 0.020). Time estimation task results were not correlated to objective SOLs (Spearman rho = 0.20, p = 0.32) or to ISI scores (Pearson r = −0.16, p = 0.43).

### 3.3 Pre Sleep Arousal Scale

**Fig. 4:**
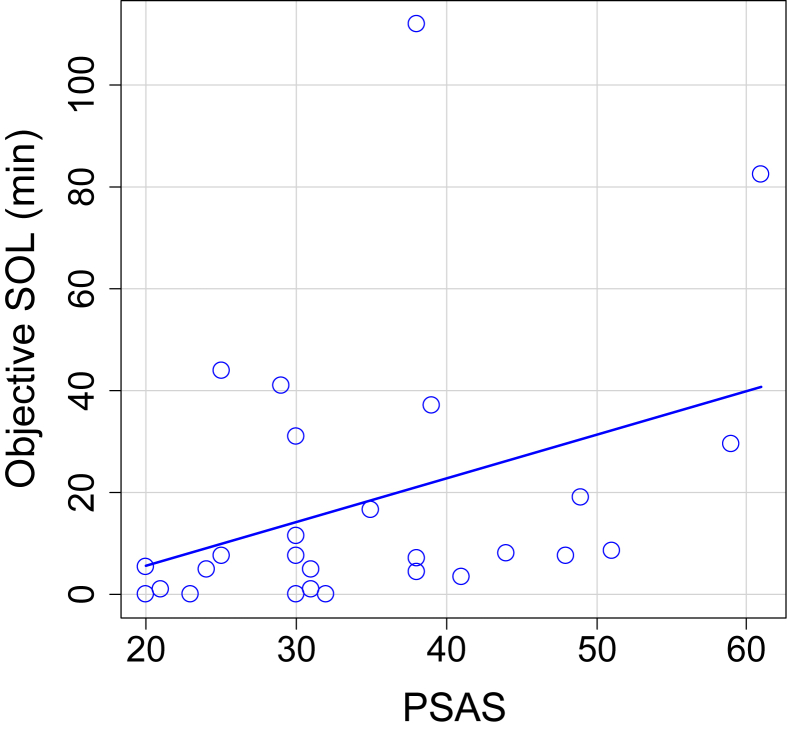
Correlation between PSAS and objective SOL (n = 27; Spearman rho = 0.41, p = 0.035).

**Fig. 5:**
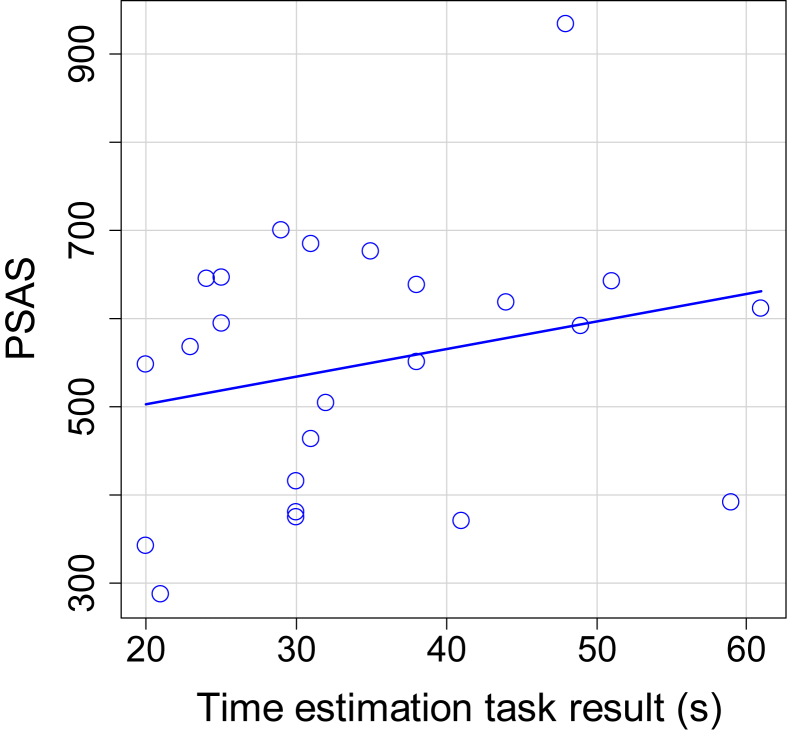
Correlation between time estimation task results and PSAS scores (n = 26; Pearson r = 0.39, p = 0.051).

The average PSAS score was 20.44 ± 7.85 [8–27]. No significant correlation was found between PSAS scores and the residual error of the sleep length model (Spearman rho = −0.13, p = 0.53, Fig. 3b). PSAS was correlated with objective SOL (Spearman rho = 0.41, p = 0.035; Fig. 4). The PSAS scores were not significantly correlated to the results of the time estimation task (Pearson r = 0.39, p = 0.051; Fig. 5).

## 4 Discussion

Our goal was to obtain a clearer view of the association of time estimation and pre sleep arousal with the amount of sleep onset misperception, while taking interindividual variation of sleep architecture into account. We estimated the perceived SOL of individual participants from the hypnogram, using model parameters obtained from a previously proposed model of the influence of sleep fragmentation on sleep onset misperception [18]. Subsequently, we calculated the residual error between estimated SOL and actually perceived SOL for each participant. This approach allowed to specifically examine the components of sleep onset misperception not explained by sleep fragmentation (ie, the residual error of the model). We found a correlation between the residual error of the model and the results of a time estimation task. A correlation between the residual error of the model and PSAS scores was not found.

The correlation between time estimation task results and sleep onset misperception had a negative coefficient, as was expected from the design of the task. Importantly, on average the time estimation of our participants was almost 10% too short. As opposed to this, in a similar study design, Harrow et al., found that both people with insomnia and healthy controls were very accurate on the time estimation task [4]. Although group differences were not significant, healthy people showed a tendency to estimate a longer time than insomnia patients [4]. Thus, our insomnia patients scored worse compared to both the insomnia and healthy controls reported form earlier research. This difference might be explained by the severity of the insomnia complaints, since the participants of our study were treatment-seeking people with insomnia who were referred to a tertiary sleep center. As a contrast, in most other time estimation task protocols volunteers with insomnia complaints were recruited from the general population [4,6]. A question that arises from our results is whether time estimation and sleep architecture independently influence sleep onset misperception, or if time estimation somehow modifies the reaction of the sleep perception of an individual to the presence of short sleep fragments. This question could potentially be answered using more advanced statistical models in a larger dataset.

Although pre sleep arousal is one of the key features of insomnia, the hypothesis that pre sleep arousal is specifically involved in sleep onset misperception remains to be confirmed. In our study, the absence of a correlation between PSAS and sleep onset misperception not explained by sleep fragmentation, together with the presence of a correlation of PSAS with objective sleep onset, points towards pre sleep arousal being more involved in sleep architecture than in the perception of the sleep. This finding is not in line with a previous study, which indicate that PSAS does play a role in subjective but not objective sleep onset [11]. A possible explanation for this difference is that the interview completed by van Egeren et al. [11], is a more precise approximation of current pre sleep arousal of the participants compared to the PSAS questionnaire, because the interview was performed on the day of the sleep recording. Another intriguing possibility is that an increased level of arousal while falling asleep might contribute to sleep onset misperception by altering the architecture of the sleep at the beginning of the night, instead of altering an individual's sensitivity for the presence of short sleep fragments. This might be an interesting subject for further research.

Both the time estimation task and the PSG recordings were performed at home, giving the participants the opportunity to freely choose their bedtimes and making the data more generalizable to daily circumstances. A disadvantage of our protocol was the lack of standardization of the time of the day and the time of the year in which the time estimation task was done. Because of practical considerations, time estimation tasks were performed between 10:00 in the morning and 19:30 in the evening. Although this design does eliminate circadian effects, it could also potentially cause variation between subjects. However, Harrow et al., did not find differences between result of the time estimation tasks performed during daytime and nighttime [4].

In a recent study, we stated that the SFPI can be regarded as a measure of sensitivity of an individual's sleep onset perception to sleep fragmentation [18]. As such, the SFPI could be correlated with time estimation and pre sleep arousal. However, calculating SFPIs from a single night of PSG poses two practical difficulties. First, typically not all possible lengths of sleep fragments are available at sleep onset during one night. Therefore, SFPIs cannot be calculated precisely and are sometimes rough approximations, which are more useful for comparing groups than for assessing the sleep behavior of individual patients. This problem could be solved by recording multiple nights of PSG for each patient. However, PSG is a costly and obtrusive method. Second, as the model is based on the assumption that short sleep fragments are overlooked, and because we defined objective sleep onset as the first epoch scored as sleep, the model does not present an explanation for people who reported falling asleep before the objective sleep onset occurs. These model assumptions imply that the SFPI is always larger than zero, resulting in an SFPI of 0.5 for all participants who underestimate their SOL. However, a difference between a small underestimation and a large underestimation of SOL might be relevant for the correlation with the time estimation task. As an alternative approach, we estimated the sleep onset for each participant as the start of the first uninterrupted sleep fragment longer than 30 min. The prediction error of the model was then used to express the unexplained component of sleep onset misperception. The choice of assigning an SFPI value of 30 min to each study participant was made because the median optimum parameter for insomnia patients was approximately 30 min in previous research [14,18].

The results of this study represent a next step towards a better understanding of the underlying mechanisms of sleep onset misperception. As far as we are aware, it is not clear whether misperception of sleep onset and of TST and wake after sleep onset have the same underlying mechanisms. Since sleep onset misperception can be seen as a misperception of time awake instead of time asleep, it is possible that different mechanisms play a role. For example, we can speculate that time estimation is more important for sleep onset misperception than for TST misperception, because time estimation tasks are performed during wake. This remains to be further investigated.

From the current results, it appears that sleep onset misperception can be partly explained by a combination of objectively measurable sleep architecture and time estimation ability of the individual. Since all people have a certain degree of sleep fragmentation, which most probably differs between nights, it is plausible that the majority of people has some amount of sleep onset misperception now and then. We found a large range of time estimation abilities within the insomnia group, co-occurring with a range of sleep onset misperception. As such, it seems plausible that sleep onset misperception is a generic characteristic of insomnia. Therefore, identifying mechanisms of sleep onset misperception could be valuable for the understanding of the pathophysiology of insomnia in general. At the same time, we do not rule out the possibility that individuals with a lot of sleep onset misperception may be a subgroup with different etiology. Time estimation and sleep onset misperception were not correlated with ISI scores, indicating that the perceived severity of insomnia probably was influenced by other factors, for example misperception of TST, an objective short sleep duration, or complaints of reduced functioning during the day. It is possible that combinations of different psychological and physiological mechanisms result in different subtypes of insomnia, requiring different types of treatment. Thus, increased knowledge about sleep onset misperception may have important consequences for the selection and tailoring of treatment, including the identification of factors that can be specifically targeted by cognitive behavioral therapy in appropriate subgroups.

## CRediT authorship contribution statement

**Lieke W.A. Hermans:** Conceptualization, Investigation, Writing - original draft. **Marina M. Nano:** Investigation, Writing - review & editing. **Tim R. Leufkens:** Conceptualization, Resources, Writing - review & editing. **Merel M. van Gilst:** Conceptualization, Writing - review & editing. **Sebastiaan Overeem:** Conceptualization, Writing - review & editing, Supervision.

## Disclosure statement

Financial arrangements or connections that are pertinent to the submitted manuscript.

This work has been done in the IMPULS framework of the Eindhoven MedTech Innovation Center (e/MTIC, incorporating Eindhoven University of Technology, Philips Research, Sleep Medicine Center Kempenhaeghe). The funders had no role in the study design, decision to publish, or preparation of the manuscript. Philips provided support in the form of a salary for author T.L., but did not have any additional role in the study design, data collection and analysis, decision to publish, or preparation of the manuscript.

This activity is in part funded by the PPS program research and innovation of the Dutch ministry of Economic affairs and Climate.
